# Inpatient screening for albuminuria and retinopathy to predict long-term mortality in type 2 diabetic patients: a retrospective cohort study

**DOI:** 10.1186/s13098-017-0229-x

**Published:** 2017-05-03

**Authors:** Ya-Mei Hsieh, Wen-Jane Lee, Wayne H.-H. Sheu, Yu-Hsuan Li, Shih-Yi Lin, I.-Te Lee

**Affiliations:** 1 Division of Endocrinology and Metabolism, Department of Internal Medicine, Taichung Veterans General Hospital, No. 1650 Taiwan Boulevard, Sect. 4, Taichung, 40705, Taiwan; 2 Department of Medical Research, Taichung Veterans General Hospital, Taichung, 40705 Taiwan; 3 School of Medicine, National Yang-Ming University, Taipei, 11221 Taiwan; 4 School of Medicine, Chung Shan Medical University, Taichung, 40201 Taiwan; 5 Center for Geriatrics and Gerontology, Taichung Veterans General Hospital, Taichung, 40705 Taiwan

**Keywords:** Albuminuria, Inpatient, Mortality, Retinopathy

## Abstract

**Background:**

There is a high hospitalization rate for diabetic patients. Since retinopathy and albuminuria are both important manifestations of microvascular disease in diabetes, our aim was to investigate the effect of retinopathy and albuminuria on long-term mortality in type 2 diabetic inpatients through this observational cohort study.

**Methods:**

Type 2 diabetic inpatients given a primary diagnosis of poor glucose control were consecutively enrolled during their hospitalization periods. Clinical information was collected through review of each patient’s medical records, and mortality data were obtained from the national registry in Taiwan.

**Results:**

A total of 761 type 2 diabetic inpatients were enrolled in the study with a median follow-up period of 6.6 years (interquartile range, 4.0–9.6 years). Patients in the Albuminuria(−)/Retinopathy(+), Albuminuria(+)/Retinopathy(−) and Albuminuria(+)/Retinopathy(+) groups had significantly higher risks of all-cause mortality and cardiovascular mortality than those in the Albuminuria(−)/Retinopathy(−) group. However, among patients with albuminuria, there was no significant difference in cumulative mortality between those with and without retinopathy (P = 0.821). A decrease in the estimated glomerular filtration rate (eGFR), but not retinopathy, was an independent predictor of all-cause mortality (95% CI 0.647‒0.893; P < 0.001) and cardiovascular mortality (95% CI 0.564‒0.921; P = 0.009) in type 2 diabetic inpatients with albuminuria.

**Conclusions:**

Albuminuria in type 2 diabetic inpatients is a strong predictor of long-term mortality after discharge from the hospital. Retinopathy is an independent predictor of mortality in type 2 diabetic inpatients without albuminuria but not in those with albuminuria. A low eGFR is a better predictor of mortality than retinopathy in type 2 diabetic inpatients with albuminuria.

## Background

Diabetes mellitus is a complex metabolic disorder, and poor blood glucose control is associated with sequential chronic microvascular complications [1]. Albuminuria not only indicates the presence of diabetic nephropathy but also predicts mortality [1–4]. Therefore, early identification and prevention of albuminuria plays a vital part in the clinical management of diabetes [5].

In addition to nephropathy, retinopathy is another significant manifestation of microvascular disease in subjects with diabetes [1]. A high prevalence of diabetic retinopathy has been reported in diabetic patients with albuminuria [6, 7], and the mortality rate of diabetic patients diagnosed with both retinopathy and albuminuria is high [8]. Awareness of the presence of retinopathy is essential in all diabetic patients, not only in those with albuminuria [1, 9]. However, the frequency of eye examinations and level of eye care are low in outpatient practice for diabetic patients [10].

Since there is a high hospitalization rate in subjects with diabetes [11], screening for albuminuria and retinopathy is practical for inpatients with diabetes. The long-term mortality rate of diabetic inpatients with albuminuria or retinopathy after discharge from the hospital has seldom been investigated. Therefore, we assessed the impact of albuminuria and retinopathy on long-term mortality in diabetic patients hospitalized due to poor blood glucose control.

## Methods

### Subjects

This study was conducted in the Endocrinology and Metabolism ward of Taichung Veterans General Hospital. Data collection was performed through the review of medical records of the diabetic patients hospitalized between August 1, 1996 and August 31, 2007. In general, all hospitalized type 2 diabetic patients, along with type 1 patients with a diabetic duration of more than 5 years, underwent urine collection and ophthalmology consultation for the evaluation of microvascular complications before being discharged. Patients were included in the study if (1) they were admitted due to a primary diagnosis of poor glucose control, (2) they had undergone ophthalmology consultation, and (3) urinary albumin excretion and serum creatinine had been assessed. Patients were excluded from analyses if (1) there were any inconsistent interpretations of eye assessments during hospitalization, (2) they had been hospitalized in critical condition, with a systolic blood pressure lower than 80 mmHg, or (3) they had died in the hospital. In the case of patients who had been hospitalized more than once during the study period, only the records of their last admission were analyzed.

### Assessments

Mortality data up to December 31, 2011 were obtained from the Collaboration Center of Health Information Application, Department of Health, Executive Yuan, Taiwan. This study complied with the tenets of the Declaration of Helsinki, and the research protocol was approved by the Institutional Review Board of Taichung Veterans General Hospital.

Based on the standard procedure in our ward during this period, all fundoscopic data were reviewed by ophthalmologists. If any abnormal findings were discovered by their fundoscopic assessments, retinal angiography (CF-60UVi fundus camera, Canon, Japan) was performed to confirm a retinopathy diagnosis. Patients were excluded from the analysis if the interpretations were inconsistent between fundoscopy and angiography. In the present study, we defined the presence of diabetic retinopathy including non-proliferative diabetic retinopathy (NPDR) and proliferative diabetic retinopathy (PDR) [12].

Laboratory analyses were performed according to the standard procedures of our ward. In brief, blood samples for biochemistry analyses were collected after an overnight fast. HbA1c was determined by cation-exchange high-pressure liquid chromatography (NGSP certificated; G8, TOSOH, Tokyo, Japan). Serum levels of total cholesterol, high-density lipoprotein (HDL) cholesterol and triglycerides were determined using enzymatic methods (Advia 1800, Siemens, New York, U.S.A.). Creatinine levels were determined using the Jaffé method (Advia 1800, Siemens, New York, U.S.A.), and urinary albumin levels were determined using the polyethylene glycol enhanced immuno turbidimetric method (Advia 1800, Siemens, New York, U.S.A.). The calculation of estimated glomerular filtration rate (eGFR) was applied by 186 × [serum creatinine (mg/dL)]^−1.154^ × [age (year)]^−0.203^ (×0.742, if female) mL/min/1.73 m^2^ based on the modification of diet in renal disease (MDRD) study equation [2]. The urine albumin creatinine ratio (ACR) was determined by the ratio of urine albumin (in milligrams) to urine creatinine (in grams). Albuminuria was defined as an ACR ≥300 mg/g [1, 2]. Hypertension was defined as a blood pressure higher than 130/80 mmHg or a history of being prescribed anti-hypertensive medications.

### Statistical analysis

Continuous data were presented as the mean ± standard deviation (SD). One-way analysis of variance (ANOVA) was used to compare the differences among groups. Pairwise multiple comparisons were performed to determine the significance of differences between two groups after ANOVA had revealed any statistical significance. Chi square test was used to compare categorical variables across the groups. The overall significance of univariate survival analysis was detected through the use of the log-rank test using Kaplan–Meier analysis. Multivariate Cox proportional hazards regression analyses were conducted to determine the hazard ratios. The statistical significance was set at P < 0.05. Statistical analysis was performed using SPSS 22.0 (IBM Corp., Armonk, NY, USA).

## Results

**Fig. 1:**
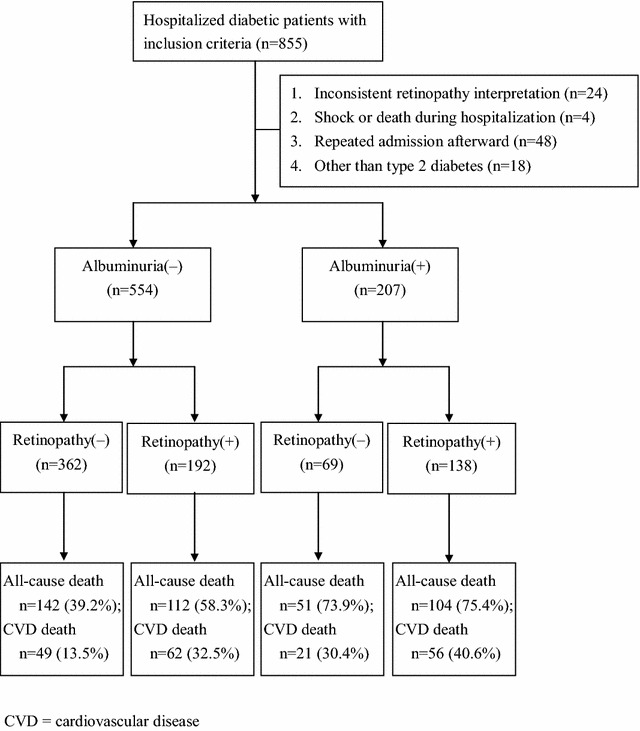
The presence of albuminuria or retinopathy during the hospitalization, and the cumulative mortality rate after discharge from the hospital

A total of 855 admissions satisfied the inclusion criteria; however, 76 admissions were excluded, including 24 admissions with inconsistent retinopathy interpretation between fundoscopy and angiography, 4 admissions in which the patients died or underwent shock, and 48 admissions after which patients were repeatedly hospitalized during the study period. Furthermore, 17 patients were excluded due to having type 1 diabetes and one patient with diabetes due to chronic pancreatitis was also excluded in the enrollment. Therefore, a total of 761 diabetic inpatients were enrolled for analyses. Based on ACR, there were 207 (27.2%) patients with albuminuria and 554 (72.8%) without albuminuria. Based on fundoscopic examination, there were 330 (43.4%) patients with retinopathy and 431 (56.6%) without retinopathy among all study subjects. The prevalence of retinopathy was higher in patients with albuminuria than in those without (66.7% vs. 34.7%, P < 0.001). Over a median period of 6.6 years (interquartile range, 4.0‒9.6 years) after discharge from hospital, there were 409 (53.7%) subjects who died during the follow-up period. Among the patients without albuminuria, the cumulative mortality rate of patients with retinopathy was significantly higher than that of patients without retinopathy (58.3% vs. 39.2%; P < 0.001). However, among patients with albuminuria, the cumulative mortality rate was not significantly different between those with retinopathy and those without (75.4% vs. 73.9%, P = 0.821) (Fig. 1).

**Fig. 2:**
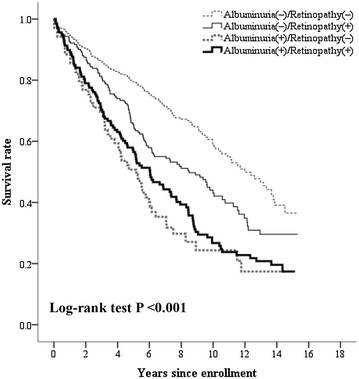
Kaplan–Meier curves showing survival rates grouped by the presence or absence of retinopathy and albuminuria

All study subjects were divided into four groups, including the patients with neither albuminuria nor retinopathy [Albuminuria(−)/Retinopathy(−)], patients with retinopathy but no albuminuria [Albuminuria(−)/Retinopathy(+)], patients with albuminuria but no retinopathy [Albuminuria(+)/Retinopathy(−)], and patients with albuminuria and retinopathy [Albuminuria(+)/Retinopathy(+)]. Table 1 shows the characteristics of these four groups. Figure 2 shows that the survival rate of the patients in the Albuminuria(−)/Retinopathy(−) group was highest among these four groups (log-rank test P < 0.001) using Kaplan–Meier analysis. Among the patients without albuminuria, the incidence of mortality in patients with retinopathy was significantly higher than that of patients without retinopathy (8.4 vs. 5.3 events/100 person-year; P < 0.001). However, among patients with albuminuria, the incidence of mortality was not significantly different between those with retinopathy and those without (11.9 vs. 14.2 events/100 person-year; P = 0.310).

**Table 1:** The clinical data of patients according to the presence or absence of albuminuria and retinopathy

	Albuminuria(−)/Retinopathy(−) (n = 362)	Albuminuria(−)/Retinopathy(+) (n = 192)	Albuminuria(+) Retinopathy(−) (n = 69)	Albuminuria(+) Retinopathy(+) (n = 138)	P
Age (years)	61 ± 15	66 ± 12	68 ± 12	65 ± 11	*<0.001*
Male, n (%)	224 (61.9%)	98 (51.0%)	37 (53.6%)	70 (50.7%)	*0.034*
BMI (kg/m^2^)	24.1 ± 4.1	23.6 ± 4.2	24.4 ± 3.8	24.1 ± 4.0	0.528
Systolic blood pressure (mmHg)	127 ± 16	130 ± 15	137 ± 16	142 ± 15	*<0.001*
Diastolic blood pressure (mmHg)	75 ± 11	74 ± 10	77 ± 10	79 ± 10	*<0.001*
Diabetes duration (years)	6.9 ± 6.7	12.3 ± 7.8	10.5 ± 8.2	12.3 ± 7.3	*<0.001*
Current smoker, n (%)	109 (30.1%)	48 (25.0%)	21 (30.4%)	35 (25.4%)	0.506
White blood cell count (10^6^/L)	8045 ± 3199	8193 ± 3565	8850 ± 3988	8389 ± 2917	0.412
HbA1c (%)	11.1 ± 3.1	10.3 ± 2.7	10.7 ± 2.9	9.3 ± 2.4	*<0.001*
Total cholesterol (mmol/L)	4.8 ± 1.5	4.7 ± 1.3	5.0 ± 1.5	5.4 ± 1.8	*<0.001*
Triglyceride (mmol/L)	2.2 ± 2.8	1.7 ± 1.2	2.1 ± 1.4	2.3 ± 2.6	*0.044*
HDL cholesterol (mmol/L)	1.0 ± 0.3	1.1 ± 0.4	1.0 ± 0.4	1.0 ± 0.4	0.127
eGFR (mL/min/1.73 m^2^)	73 ± 33	68 ± 29	52 ± 28	53 ± 30	*<0.001*
Albumin to creatinine ratio (mg/g)	54 ± 64	98 ± 83	1226 ± 1285	2825 ± 5043	*<0.001*
Hypertension, n (%)	246 (68.0%)	145 (75.5%)	57 (82.6%)	125 (90.6%)	*<0.001*
Antihypertensive agents, n (%)	165 (45.6%)	110 (57.3%)	40 (58.0%)	81 (58.7%)	*0.009*
ACE inhibitor or ARB, n (%)	110 (30.4%)	68 (35.4%)	25 (36.2%)	61 (44.2%)	*0.035*
α-Blocker, n (%)	47 (13.0%)	26 (13.5%)	11 (15.9%)	13 (9.4%)	0.545
β-Blocker, n (%)	20 (5.5%)	17 (8.9%)	5 (7.2%)	10 (7.2%)	0.520
Calcium channel blocker, n (%)	66 (18.2%)	51 (26.6%)	27 (39.1%)	39 (28.3%)	*<0.001*
Diuretics, n (%)	20 (5.5%)	17 (8.9%)	9 (13.0%)	22 (15.9%)	*0.002*
Oral antihyperglycemic drugs, n (%)	158 (43.6%)	75 (39.1%)	31 (44.9%)	37 (26.8%)	*0.005*
Insulin secretagogues, n (%)	139 (38.4%)	64 (33.3%)	29 (42.0%)	34 (24.6%)	*0.018*
Metformin, n (%)	84 (23.2%)	48 (25.0%)	16 (23.2%)	20 (14.5%)	0.117
Thiazolidinediones, n (%)	7 (1.9%)	4 (2.1%)	0 (0.0%)	0 (0.0%)	0.242
α-Glucosidase inhibitor, n (%)	6 (1.7%)	4 (2.1%)	1 (1.4%)	0 (0.0%)	0.443
Insulin therapy, n (%)	224 (61.9%)	99 (51.6%)	35 (50.7%)	42 (30.4%)	*<0.001*
Statins, n (%)	39 (10.8%)	18 (9.4%)	4 (5.8%)	15 (10.9%)	0.616

Statistical significance (P < 0.05) is indicated in italics

*ACE* angiotensin-converting enzyme, *ARB* angiotensin II receptor blocker, *BMI* body mass index, *eGFR* estimated glomerular filtration rate, *HbA1c* glycated hemoglobin, *HDL* high-density lipoprotein

During the follow-up period, Cox regression analysis showed that patients in the Albuminuria(−)/Retinopathy(−) group had the lowest risk for all-cause mortality and cardiovascular mortality. Patients in the Albuminuria(−)/Retinopathy(+) group had significantly higher all-cause mortality (hazard ratio [HR] = 1.524, 95% confidence interval [CI] 1.138–2.041) and cardiovascular mortality (HR = 2.434, 95% CI 1.562–3.794) than patients in the Albuminuria(−)/Retinopathy(−) group. Patients in the Albuminuria(+)/Retinopathy(−) group had significantly higher all-cause mortality (HR = 2.551, 95% CI 1.771–3.676) and cardiovascular mortality (HR = 2.762, 95% CI 1.551–4.917), and patients in the Albuminuria(+)/Retinopathy(+) group also had significantly higher all-cause mortality (HR = 2.200, 95% CI 1.613–3.001) and cardiovascular mortality (HR = 3.327, 95% CI 2.080–5.321) than patients in the Albuminuria(−)/Retinopathy(−) group. These conclusions were made after data were adjusted for age, gender, body mass index, systolic blood pressure, current smoker, diabetes duration, HbA1c, chronic kidney disease, total cholesterol, triglycerides, insulin treatment, and angiotensin-converting enzyme inhibitor/angiotensin II receptor blocker treatment (Table 2).

**Table 2:** Results of Cox regression analysis for the effects of albuminuria and retinopathy on (A) all-cause and (B) cardiovascular mortality

	Crude			Crude			Crude			Crude		
	HR	95% CI	P	HR	95% CI	P	HR	95% CI	P	HR	95% CI	P
(A) All-cause-mortality												
Albuminuria(−)/Retinopathy(−)	1			1						1		
Albuminuria(−)/Retinopathy(+)	1.575	(1.230, 2.018)	*<0.001*	1.402	(1.092, 1.801)	*0.008*	1.529	(1.142, 2.047)	*0.004*	1.524	(1.138, 2.041)	*0.005*
Albuminuria(+)/Retinopathy(−)	2.714	(1.969, 3.741)	*<0.001*	2.465	(1.784, 3.405)	*<0.001*	2.550	(1.772, 3.671)	*<0.001*	2.551	(1.771, 3.676)	*<0.001*
Albuminuria(+)/Retinopathy(+)	2.241	(1.739, 2.887)	*<0.001*	2.215	(1.718, 2.856)	*<0.001*	2.213	(1.626, 3.014)	*<0.001*	2.200	(1.613, 3.001)	*<0.001*
(B) Cardiovascular mortality												
Albuminuria(−)/Retinopathy(−)	1			1			1			1		
Albuminuria(−)/Retinopathy(+)	2.519	(1.731, 3.664)	*<0.001*	2.155	(1.478, 3.144)	*<0.001*	2.412	(1.550, 3.752)	*<0.001*	2.434	(1.562, 3.794)	*<0.001*
Albuminuria(+)/Retinopathy(−)	3.264	(1.955, 5.448)	*<0.001*	2.858	(1.707, 4.785)	*<0.001*	2.866	(1.610, 5.105)	*<0.001*	2.762	(1.551, 4.917)	*<0.001*
Albuminuria(+)/Retinopathy(+)	3.489	(2.376, 5.123)	*<0.001*	3.440	(2.340, 5.057)	*<0.001*	3.339	(2.094, 5.326)	*<0.001*	3.327	(2.080, 5.321)	*<0.001*

Statistical significance (P < 0.05) is indicated in italics

*HR* hazard ratio, *CI* confidence interval

Model 1: adjusted for age and gender

Model 2: adjusted for age, gender, body mass index, systolic blood pressure, current smoker, diabetes duration, glycated hemoglobin, chronic kidney disease [estimated glomerular filtration rate (eGFR) <60 mL/min/1.73 m^2^], total cholesterol and triglycerides

Model 3: adjusted for age, gender, body mass index, systolic blood pressure, current smoker, diabetes duration, glycated hemoglobin, chronic kidney disease (eGFR <60 mL/min/1.73 m^2^), total cholesterol, triglycerides, insulin treatment and angiotensin-converting enzyme inhibitor/angiotensin II receptor blocker treatment

In the patients with albuminuria, Table 3A shows that presence of retinopathy was not a significant predictor for all-cause mortality either in univariate analyses or in multivariate analyses after being adjusted for albuminuria (both P > 0.05). However, eGFR was a significant predictor for all-cause mortality both in univariate analyses and in multivariate analyses (both P < 0.001). Similarly, in the patients with albuminuria, Table 3B shows that the presence of retinopathy was not a significant predictor for cardiovascular mortality either in univariate analyses or in multivariate analyses after being adjusted for albuminuria (both P > 0.05). ACR and eGFR were significant predictors for cardiovascular mortality both in univariate analyses (P = 0.041 for ACR and 0.013 for eGFR, respectively) and in multivariate analyses (P = 0.039 for ACR, and 0.009 for eGFR, respectively).

**Table 3:** Results of Cox regression analysis for the effects of risk factors on (A) all-cause and (B) cardiovascular mortality in patients with albuminuria

	Univariate model			Multivariate model^a^					
	Crude			Model 1			Model 2		
	HR	95% CI	P	HR	95% CI	P	HR	95% CI	P
(A) All-cause mortality									
Retinopathy (yes/no)	0.840	(0.599, 1.177)	0.310	0.850	(0.595, 1.213)	0.370	0.672	(0.409, 1.104)	0.117
Urine albumin to creatinine ratio^b^	1.167	(0.820, 1.662)	0.390	1.359	(0.950, 1.945)	0.094	1.062	(0.664, 1.699)	0.801
eGFR (every 15 mL/min/1.73 m^2^)	0.843	(0.769, 0.924)	<*0.001*				0.760	(0.647, 0.893)	*<0.001*
Current smoker (yes/no)	1.127	(0.790, 1.606)	0.509				0.890	(0.517, 1.532)	0.675
Diabetes duration (every 1 year)	1.012	(0.991, 1.033)	0.271				0.996	(0.967, 1.025)	0.781
BMI (every 1 kg/m^2^)	0.980	(0.929, 1.033)	0.453				0.981	(0.920, 1.045)	0.551
Systolic blood pressure (every 10 mmHg)	1.008	(0.906, 1.121)	0.886				0.958	(0.826, 1.110)	0.567
HbA1c (every 1%)	1.004	(0.939, 1.074)	0.895				1.024	(0.933, 1.122)	0.621
Total cholesterol (every 1 mmol/L)	0.949	(0.857, 1.050)	0.313				1.073	(0.920, 1.251)	0.368
Triglycerides (every 1 mmol/L)	0.985	(0.917, 1.058)	0.677				0.987	(0.908, 1.073)	0.762
(B) Cardiovascular mortality									
Retinopathy (yes/no)	1.111	(0.671, 1.840)	0.683	1.074	(0.632, 1.825)	0.793	0.937	(0.433, 2.028)	0.868
Albumin to creatinine ratio^b^	1.636	(1.021, 2.623)	*0.041*	1.839	(1.141, 2.965)	*0.012*	1.965	(1.035, 3.730)	*0.039*
eGFR (every 15 mL/min/1.73 m^2^)	0.849	(0.745, 0.966)	*0.013*				0.721	(0.564, 0.921)	*0.009*
Current smoker (yes/no)	1.222	(0.745, 2.004)	0.428				1.039	(0.429, 2.518)	0.932
Diabetes duration (every 1 year)	1.029	(1.000, 1.059)	*0.049*				1.011	(0.969, 1.055)	0.610
BMI (every 1 kg/m^2^)	1.003	(0.931, 1.080)	0.942				1.010	(0.918, 1.110)	0.843
Systolic BP (every 10 mmHg)	1.041	(0.876, 1.237)	0.645				0.974	(0.776, 1.224)	0.824
HbA1c (every 1%)	1.021	(0.921, 1.133)	0.689				1.049	(0.908, 1.212)	0.515
Total cholesterol (every 1 mmol/L)	0.861	(0.693, 1.071)	0.179				0.952	(0.744, 1.219)	0.697
Triglycerides (every 1 mmol/L)	0.946	(0.748, 1.197)	0.645				1.003	(0.883, 1.139)	0.967

Statistical significance (P < 0.05) is indicated in italics

*BMI* body mass index, *eGFR* estimated glomerular filtration rate, *HbA1c* glycated hemoglobin, *CI* confidence interval, *HR* hazard ratio, *BP* blood pressure

^a^ After adjusted for age and gender

^b^ Because of the skewed distribution, the albumin to creatinine ratio was logarithm-transformed (log) for the analyses

## Discussion

Diabetic retinopathy is associated with systemic vascular inflammation [13, 14], and it increases the risk of mortality [9, 15, 16]. Despite a high mortality rate in the patients with both retinopathy and macroalbuminuria, the presence of retinopathy did not significantly increase the risk of all-cause or cardiovascular mortality in the subgroup of normoalbuminuria in a Chinese diabetic population [8]. Similarly, in an analysis based on the data from a National Health and Nutrition Examination Survey (NHANES), it was found that presence of retinopathy did not significantly predict total mortality in the subgroup of the patients without albuminuria [17]. However, based on fundoscopic screens during hospitalization, our results showed that retinopathy increased the risk of long-term mortality by 52.4% in type 2 diabetic inpatients without albuminuria. Since retinopathy is not rare in diabetic patients without albuminuria [18, 19], routine ophthalmology consultation is strongly recommended for diabetic inpatients, especially for those without albuminuria [20].

Previous research has shown that albuminuria is a predictor of mortality in Asian as well as Caucasian diabetic patients [21, 22]. Recently, Aragón-Sánchez et al. [23] reported that albuminuria was associated with in-hospital deaths of diabetic patients with foot complications. In the present cohort, over a median period of 6.6 years after discharge from hospital, the type 2 diabetic patients with albuminuria had a higher risk of all-cause and cardiovascular mortality than those without albuminuria detected during hospitalization. This result outlines the importance for assessment of albuminuria in hospitalized type 2 diabetic patients.

There is a high prevalence of retinopathy among diabetic patients with albuminuria [24]. Therefore, the impact of retinopathy on mortality could be attributed to its interaction with albuminuria [8, 25]. It has been reported that albuminuria is a stronger predictor of mortality than retinopathy and that retinopathy is not an independent predictor of mortality after adjusting for the presence of albuminuria in the diabetic population [25]. Our findings provide evidence that retinopathy is a significant predictor of mortality in type 2 diabetic inpatients without albuminuria but not in those with albuminuria. It is noteworthy that a low eGFR, but not the presence of retinopathy, was an independent predictor of mortality in type 2 diabetic inpatients with albuminuria in the present study. Bello et al. also showed that retinopathy could not predict all-cause mortality in diabetic patients with chronic kidney disease [26]. Since eGFR and albuminuria showed a synergistic effect on mortality [27, 28], the calculation of eGFR is important for type 2 diabetic patients with albuminuria during hospitalization.

The mortality rate of the diabetic population is 1.63-fold higher in comparison to the general population based on the data from the National Register of Deaths [29]. Furthermore, the mortality rate of hospitalized diabetic patients was 2.98-fold higher than that of Taiwan’s general population [30]. Although the mortality rate of diabetic patients has decreased in recent decades [31], there was still a high cumulative mortality rate (53.7%) in type 2 diabetic inpatients admitted due to poor blood glucose control in the present study. Particularly in the type 2 diabetic inpatients with albuminuria, the mortality rate reached as high as 74.9% following a median time of 6.6 years upon discharge. Lipska et al. also found a high mortality rate of approximately 17% in diabetic inpatients within one year of discharge from the hospital [11]. The predictive factors of mortality for diabetic inpatients are emergent, and require further investigation.

Screening for retinopathy and nephropathy in diabetic patients is mostly done in the outpatient department. In view of the high mortality rate in diabetic inpatients admitted for poor glucose control, it is essential to routinely assess these predictors of mortality during hospitalization. However, evidence of the value of screening for retinopathy and albuminuria in hospitalized diabetic patients has not previously been adequately presented. Although various causes may bring about hyperglycemia in hospitalized type 2 diabetic patients, our findings suggest that routine screening for retinopathy and urine albumin excretion can be helpful in predicting mortality after discharge. Furthermore, as noted in a previous report, approximately 38% of hospitalized patients have diabetes, and more than 30% of those are newly diagnosed cases [32]. Therefore, inpatient assessment of retinopathy and nephropathy is also useful in the early detection of diabetes-associated complications [33].

There were some limitations in the present study. First, there were daily variations in urinary albumin excretion, so one should not rely on a single measurement [34]. However, we used only the ACR that was detected once during the hospitalization in this study. Second, we did not take into consideration any treatment that the patients received after discharge. Third, our cohort included only type 2 diabetic patients admitted with a primary diagnosis of poor blood glucose control, meaning our findings cannot be generalized for all diabetic patients.

## Conclusions

The presence of albuminuria is an important predictor of long-term mortality in type 2 diabetic inpatients admitted due to poor blood glucose control. In type 2 diabetic inpatients without albuminuria, the presence of retinopathy is significantly associated with a higher long-term mortality. In type 2 diabetic inpatients with albuminuria, a low eGFR, but not the presence of retinopathy, is significantly associated with higher long-term mortality. In view of the increased risk of mortality after discharge, it is important to screen all hospitalized type 2 diabetic patients for nephropathy and retinopathy.

## Abbreviations

ACE: angiotensin-converting enzyme
ACR: albumin creatinine ratio
ANOVA: analysis of variance
ARB: angiotensin II receptor blocker
BMI: body mass index
CI: confidence interval
eGFR: estimated glomerular filtration rate
HbA1c: glycated hemoglobin
HDL: high-density lipoprotein
HR: hazard ratio
MDRD: modification of diet in renal disease
NPDR: non-proliferative diabetic retinopathy
PDR: proliferative diabetic retinopathy
SD: standard deviation
